# Effects of In Situ Porous Carbon Modification on Thermal Energy Storage of Paraffin/Expanded Vermiculite Form-Stable Composite Phase Change Materials

**DOI:** 10.3390/ma18040870

**Published:** 2025-02-17

**Authors:** Huijing Chen, Shaogang Zhang, Yixiu Xin, Jiaqing Zhao, Jinhong Li, Xin Min, Xiaoguang Zhang

**Affiliations:** 1 Beijing Key Laboratory of Materials Utilization of Nonmetallic Minerals and Solid Wastes, National Laboratory of Mineral Materials, School of Materials Science and Technology, China University of Geosciences, Beijing 100083, China; cchenhuijing@163.com (H.C.); zhangsg@cugb.edu.cn (S.Z.); xinyixiu99@163.com (Y.X.); jqzhao2025@163.com (J.Z.); minx@cugb.edu.cn (X.M.); 2 College of Materials Science and Engineering, Beijing University of Technology, No. 100 Pingleyuan Street, Chaoyang District, Beijing 100124, China; zhangxg@bjut.edu.cn

**Keywords:** form-stable phase change materials, paraffin, in situ porous carbon, non-isothermal crystallization study, vermiculite

## Abstract

The utilization of form-stable phase change materials (PCMs) represents a reliable technology for achieving energy conversion. In this study, starch was impregnated into the layers of expanded vermiculite (EVM) and subsequently carbonized at high temperatures to produce in situ carbon layers modified materials (EVMC), which enhance heat storage efficiency. The EVMC, characterized by its carbon network, acted as encapsulated material, leading to the development of paraffin (P)/EVCM-based shape-stable composite PCM (EVMCP). The latent heat of the EVMCP was measured at 179 J/g, surpassing that of EVMP at 144.8 J/g. This finding suggested that in situ porous carbon significantly improves the heat storage ability. Furthermore, non-isothermal crystallization curves indicated that EVMC markedly accelerated the nucleation and simultaneously restricted the non-isothermal crystal growth.

## 1. Introduction

Energy and materials have played a significant role in social development since human society entered the industrial age [1]. However, given the explosive growth of industry, energy consumption has emerged as a critical limitation to sustainable development [2]. Therefore, it is essential to develop new functional materials that exhibit high-efficiency energy conversion and possess specific properties for effective thermal management [3]. PCMs store energy by absorbing or releasing significant amounts of heat during their phase transition processes [4]. This technology offers several advantages, including high energy storage density [5], the ability to absorb or release energy within a narrow temperature range [6], and minimal volume changes during phase transitions [7]. The integration of phase change energy storage technology with existing energy systems aims to enhance energy efficiency and promote conservation efforts [8].

Solid–liquid phase change materials exhibit favorable phase change temperatures, high latent heat, and minimal volume changes. Consequently, it is extensively utilized in practical applications within the realm of thermal energy storage [9]. Specifically, paraffin has been extensively researched and utilized as a solid–liquid organic phase change material due to its low cost, high energy storage density, and non-toxicity [10]. However, paraffin presents challenges such as low thermal conductivity and leakage during the phase transition process, which diminish energy storage efficiency and constrain practical applications [11]. In light of the low thermal conductivity exhibited by phase change materials, recent studies have demonstrated that a variety of thermal conductivity enhancers, such as carbon nanoplatelets [12,13], carbon nanotubes [14], and metal oxide nanoparticles, can be incorporated into materials [15,16,17]. Furthermore, combining these enhancers with porous mineral substrates allows for the creation of composite phase transition materials that exhibit improved heat conductivity and enhanced stability. However, the thermally conductive filler is associated with high cost, unstable properties, and a tendency to accumulate, which ultimately leads to the separation of the thermal conduction pathway. Additionally, in recent years, porous mineral materials have been used to prepare morphologically stable composite PCMs, owing to their surface tension and capillary force. These materials have been extensively reported in papers [18,19,20], including expanded graphite [21,22], kaolin [23,24], Diatomite [25], expanded perlite [26], and expanded vermiculite [27], which are all used to prevent leakage of PCMs.

Expanded vermiculite (EVM) is a porous silicate mineral material characterized by its high porosity and large specific surface area [28,29], which contribute to an enhanced heat storage capacity [30]. EVM also possesses several advantages, including favorable physical properties, large reserves, and low cost, making it one of the most economical and suitable encapsulated material [31,32]. Nevertheless, it is important to note that EVM exhibits low thermal conductivity, a limitation that persists in composite phase change materials [33]. Therefore, in the investigation of expanded vermiculite as the matrix for phase change materials, Wang et al. [34] and Song et al. [35] utilized polyethylene glycol (PEG) as the phase change material, and the loading capacity of PEG was enhanced by modifying the expanded vermiculite with dopamine and acid, respectively. Yang et al. [36] modified the expanded vermiculite with acid and organic intercalation to improve the loading capacity of PEG. Thereby, modification significantly improved the thermal energy storage performance of the composite phase change material. In summary, in the research on composite phase change materials based on expanded vermiculite, the loading capacity of phase change materials were enhanced mainly through the following methods. Firstly, phase change materials were modified to improve their adhesion with the matrix; additionally, expanded vermiculite was modified to increase its porosity to expand the load capacity of phase change materials. These modifications enhanced the thermal storage performance and thermal conductivity of the composite phase change materials.

Biomass, a carbon-rich and renewable material, can be prepared using porous carbon materials after high-temperature carbonization. Its rich pore density is conducive to the adsorption of phase change materials, and carbon materials can also improve thermal conductivity [37]. Therefore, biomass is an ideal support material after carbonization. Recent studies have shown that using watermelon [38], plant leaves [39], potatoes, and white radish [40] to prepare porous carbon carrier materials can not only increase the encapsulation rate of phase change materials but also improve the thermal conductivity of composite phase change materials. However, using untreated biomass resources to prepare porous carbon materials has high costs and complex preparation processes, and is unsuitable for large-scale production.

Among the many biomass resources, starch has great advantages in the preparation of carbon materials because it is cheap, easy to obtain, green, and renewable. Shi et al. [41] used dry yeast powder to prepare porous carbon materials at various carbonization temperatures, which revealed that the thermal conductivity was 1.93 times that of paraffin. Zhao et al. [42] introduced 5wt.% graphite to the foamed carbon to prepare a carrier material, which had the best effect for encapsulating paraffin and proved that the prepared matrix could improve the heat transfer capacity. Ji et al. [43] produced an noctadecane/porous carbon composite PCM by employing functionalized polystyrene nanoparticles as a template and soluble starch as a carbon source. The thermal conductivity increased by 266% compared to n-octadecane, reaching 0.631 W/m K. The aforementioned research on the preparation of porous carrier materials utilizing starch or yeast powder as carbon sources has substantially enhanced the performance of these materials. Although there are many types of research on the composite PCM of biomass carbonization, there are few types of research based on the modification of porous carbon structures in layer clay minerals.

Therefore, developing porous mineral carrier materials that exhibit both excellent thermal conductivity and a high loading capacity presented significative research. This study introduced starch as a porous carbon source into the interlayer of EVM and then carbonized it to prepare encapsulated materials. The prepared carrier material can effectively stabilize PCM and enhance heat transfer. The non-isothermal crystallization behavior of PCM was analyzed, and the mechanism of synergistic promotion of crystallization using porous carbon and EVM was elucidated. This study could provide some references for the crystallization analysis and matrix modification of PCM.

## 2. Materials and Methods

### 2.1. Materials

Paraffin (P, 52–54 °C, CAS:646-31-1) were acquired form Sinopharm Group Chemical reagent Co., Ltd., Shanghai, China, and corn starch (C, food grade) were all acquired from Nanjing Ganzhiyuan Sugar Co., Ltd., Nanjing, China. The EVM was gained from Ling Shou.

### 2.2. Form-Stable Composite PCM Preparation

#### 2.2.1. Preparation of In Situ Carbon Layers Expanded Vermiculite

**Figure 1:**
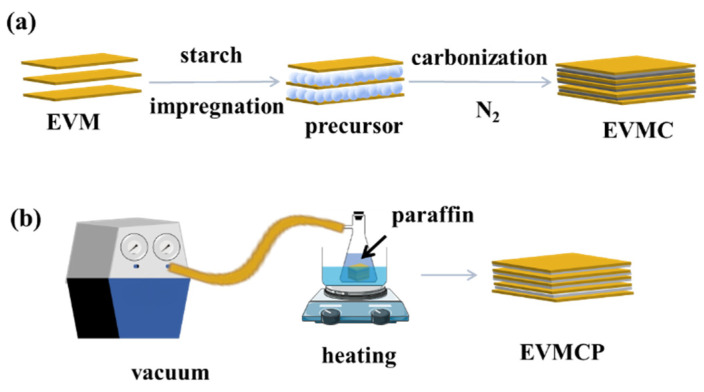
Schematic illustration of ss-CPCM preparation. (**a**) Preparation of EVMC, (**b**) Preparation of ss-CPCMs using EVM.

**Figure 2:**
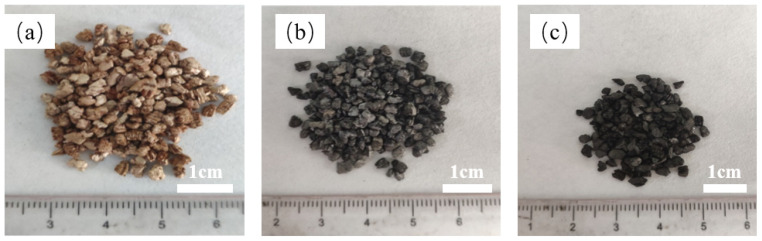
The sample picture of EVM (**a**), EVMC (**b**) and EVMCP (**c**).

The preparation process of EVMC is presented in Figure 1a. First, the expanded vermiculite was immersed in the beaker containing a 15 wt% cornstarch solution, stirred for 1 h. Subsequently, the samples were filtered out and transferred to a drying oven set at 70 °C to remove surface moisture to obtain expanded vermiculite/starch matrices. Then, the expanded vermiculite/starch matrix underwent carbonization. The temperature was gradually increased at the rate of 5 °C/min until it reached 800 °C, where it was maintained for 2 h under the nitrogen atmosphere. Finally, the expanded vermiculite/porous carbon matrix with the binary structure was obtained through natural cooling, and its morphology is shown in Figure 2b.

#### 2.2.2. Fabrication of Form-Stable Composite Phase Change Material

The preparation process of EVMCP is described in Figure 1b. The paraffin was initially placed in the vacuum filter bottle and heated in the water bath at 75 °C until it was completely melted. Next, the EVMC was introduced into the vacuum filter bottle for vacuum impregnation, and the sample was extracted following the vacuum adsorption process that lasted for 45 min at a temperature of 75 °C. Finally, the sample was placed on filter paper for repeated leakage testing to obtain EVMCP, and its morphology is displayed in Figure 2c.

### 2.3. Characterization

X-ray diffraction (XRD, D8-Advance, Bruker, Billerica, MA, USA, CuKα radiation, scan range: 8~60°, scan rate: 10.0°/min, minimum step size 0.0001°) was employed in the crystal phases of paraffin and the composite PCM. The chemical compatibility was investigated using Fourier Transform infrared spectroscopy (FT-IR, Nicolet Is10, Thermo Fisher Scientific, Waltham, MA, USA, test range: 4000 cm^−1^~400 cm^−1^, spectral resolution: better than 0.4 cm^−1^). Field emission scanning electron microscopy (FESEM, JSM-7001F, Japan Electronics Co., Ltd., Tokyo, Japan, resolution: 3 nm at 15 kV) was used to examine morphology. A differential scanning calorimeter (DSC, 214 Polyma, NETZSCH, Bavaria, Germany, temperature range: 0~80 °C, heating and cooling rate: 5 °C/min, N_2_, temperature accuracy of 0.1 °C) was utilized to determine the thermal storage capacity. A thermogravimetric analyzer (TG, STA 449 F3, NETZSCH, Bavaria, Germany, temperature accuracy of ±0.3 °C) was employed to assess the thermal stability of the materials.

A mercury intrusion meter (MIP, Micro Active Auto Pore V 9600, Micromeritics, Norcross, GA, USA, accuracy: 0.1 µL) was used to determine the pore parameters. A laser thermal conductivity tester (LFA-467, NETZSCH, Bavaria, Germany, accuracy: ±3%) was used to measure the thermal conductivity. Fourier transform infrared spectra (FTIR) were employed to describe the thermal stability of EVMCP after 100 heating–cooling cycles.

## 3. Results and Discussion

### 3.1. Pore Size Distribution Characteristics of EVM and EVMCP

**Figure 3:**
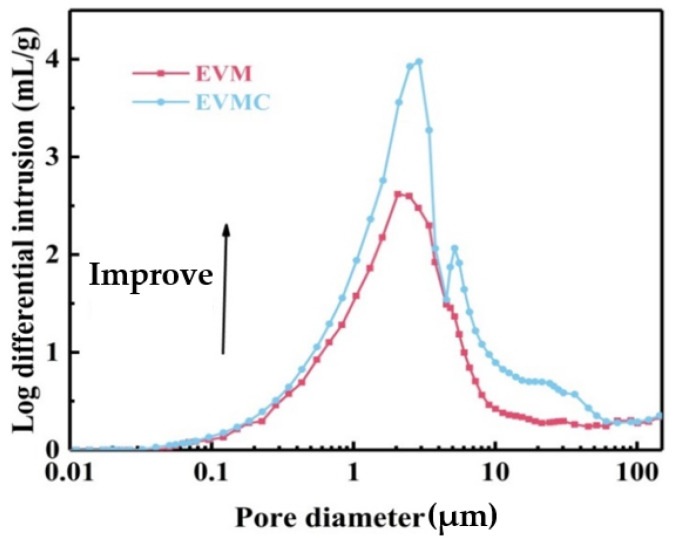
Pore structure parameters of EVM and EVMC.

Figure 3 illustrates the pore structure characteristics and significant parameters of EVM and EVMC. Porosity of EVM was 82.31%, and the average pore diameter and volume were 1.11 µm and 2.83 mL/g (Refer to Table 1), respectively. Compared to EVM’s pore size distribution properties, the introduction of porous carbon raised the incursion volume, average pore diameter, and total porosity of EVMC to 3.92 mL/g, 1.21 µm, and 89.82% (Refer to Table 1), respectively. The range of pore size dispersion was from 0.01 to 100 µm and mainly distributed between 1–10 µm. Therefore, the pores of EVMC can provide paraffin with a larger packaging capacity, which significantly increases the TES capability of EVM-based CPCMs.

### 3.2. Crystallinity Characterization of CPCMs Based on EVM

**Figure 4:**
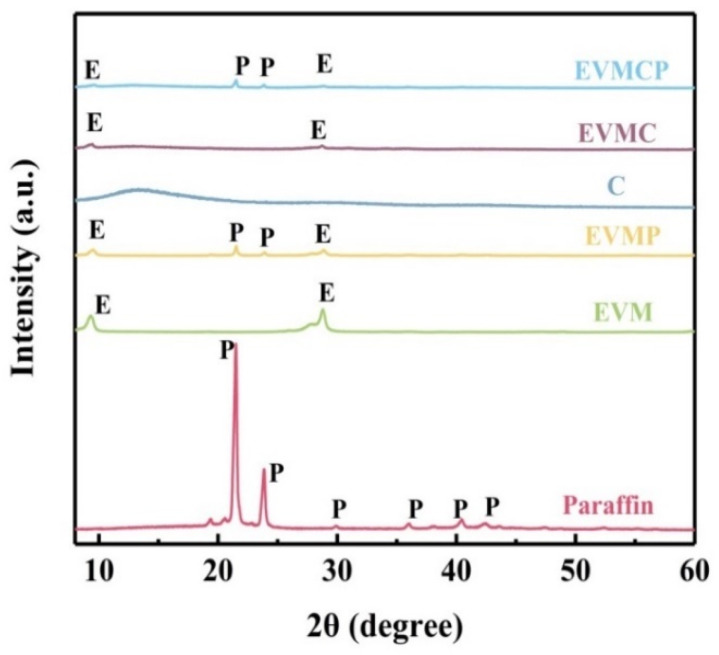
XRD patterns of samples.

Figure 4 shows P, EVMC, CPCMs’, and EVM XRD patterns. Paraffin demonstrated two strong reflections, a peak at 21.5°, and a peak at 23.9°. In the diffraction pattern of EVM, the characteristic peaks of phlogopite were identified as the diffraction peaks at 2θ = 9.3° and 28.8°. The XRD curve of porous carbon displayed broad and weak peaks. It implied that porous carbon had a low degree of graphitization. Therefore, the XRD diffraction peaks of porous carbon in EVMC and EVMCP were not obvious. There were no additional diffraction peaks found in the XRD pattern of EVMP and EVMCP, and the diffraction peaks of P and EVM were clearly apparent, indicating that the interaction of paraffin with the CPCMs based on EVM involves only physical bonding rather than chemical reaction, resulting in EVMCP’s high chemical stability.

### 3.3. FTIR Analysis

**Figure 5:**
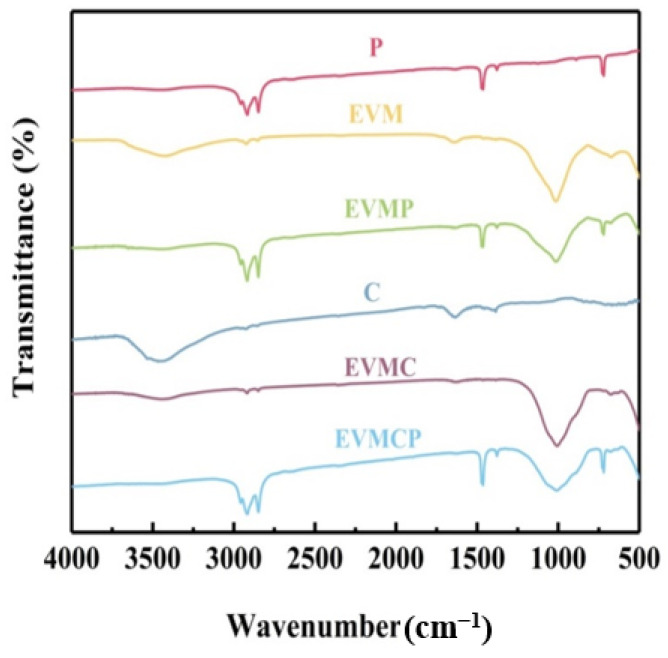
FT-IR spectra of Paraffin, EVM, C, EVMC, and the ss-CPCMs.

Figure 5 shows the FT-IR graphs of paraffin, EVM, EVMP, EVMC, C, and EVMCP. The paraffin spectrum showed absorption peaks at 2965, 2919, and 2847 cm^−1^. These were caused by the C-H stretching vibrations of -CH3 (2965 cm^−1^) and -CH_2_ (2919 and 2847 cm^−1^). Also, the absorption peaks at 1466 and 1378 cm^−1^ were linked to C-H bending vibrations in the plane, while the 719 cm^−1^ peak was linked to C-H bending vibrations that were not in the plane. The vibrations of the Si-O-M (octahedral cation) and Al-O-Si bonds matched the absorption peaks at 444 and 678 cm^−1^ in the EVM spectrum. The silicon skeleton showed Si-O stretching vibration at the peak of 998 cm^−1^. The bending and stretching vibrations of interlayer water were respectively shown at the maxima at 1637 and 3457 cm^−1^ [43]. The C-H bond stretching vibration peaks in the infrared spectrum at 2915 cm^−1^ and the bending vibration peaks of the bond at 1460 cm^−1^. This was the clearer bond structure inside the low degree of graphitization amorphous carbon. The bending and stretching vibrations of interlayer water respectively represented the peaks at 1640 and 3474 cm^−1^. The high points of EVMC matched EVM and C. Furthermore, easily observable in the spectrum of EVMP and EVMCP were the FT-IR spectra of the EVM, P, and C. Moreover, no new peaks in the spectra of the EVMP and EVMCP revealed the interaction of porous carbon. Given that EVM and paraffin created a physical relationship instead of a chemical reaction, the CPCMs showed good chemical compatibility.

### 3.4. Thermal Energy Storage Properties of CPCMs

**Figure 6:**
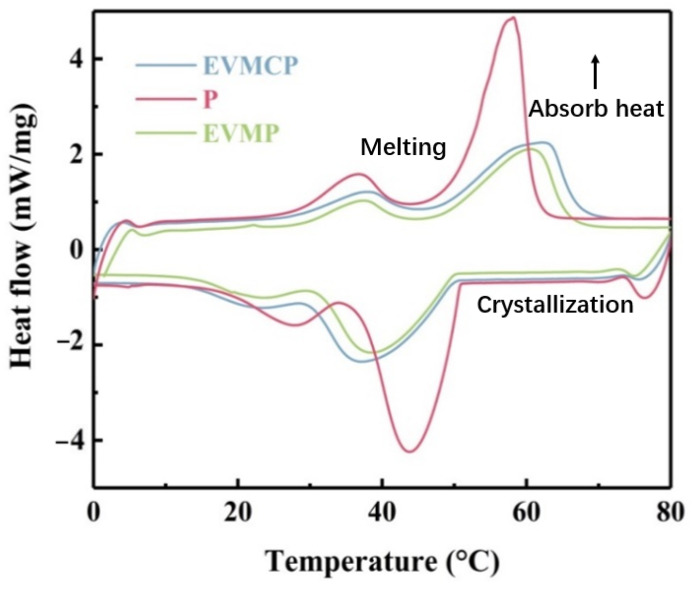
EVM-based DSC curves of Paraffin and the ss-CPCMs.

Figure 6 shows the DSC curves of paraffin and the CPCMs depending on EVM; Table 2 shows the determined phase change parameters. Table 2 and Figure 6 reveal that the DSC curves of paraffin, EVMP, and EVMCP had unambiguous endothermic and exothermic peaks with similar phase transition behavior. Whereas the solid–liquid phase change generated the second peak, the solid–solid phase transition generated the first. The melting temperature (TM) and freezing temperature (Ts) respectively referred to the solid–liquid and liquid–solid phase transition temperatures in the melting and freezing processes.

According to Table 2, the values of Tm and T_s_ of Paraffin, EVMP, and EVMCP were 58.2/43.8 °C, 60.6/38.5 °C and 62.2/37.1 °C, respectively. It was evident that the addition of porous carbon delayed the melting and freezing peak temperatures of paraffin in the phase change process. The great adsorption affinity of porous carbon to paraffin was identified as the cause of the delay in the phase transition temperatures of paraffin. Paraffin showed a high latent with the melting enthalpy of 247.2 J/g and the solidification enthalpy of 259.1 J/g. The melting and solidification latent heat values of EVMP were 144.8 J/g and 157.6 J/g, respectively. The encapsulation rate of paraffin was 59%, and the encapsulation rate of paraffin was 76%, and the melting and solidification latent heat values of EVMCP were 179 J/g and 179.2 J/g, respectively. These results demonstrated the beneficial impact of the impregnation of porous carbon. EVMCP had high enthalpy because the porous carbon was precipitated in situ among the layers of EVM with a high specific surface area and abundant microporous structure. As a result, EVMC demonstrated excellent phase change behavior. In this investigation, the prepared EVMC was chosen as the most appropriate PCMs for increasing thermal energy storage efficiency.

### 3.5. Morphology Characteristics of the ss-CPCMs

**Figure 7:**
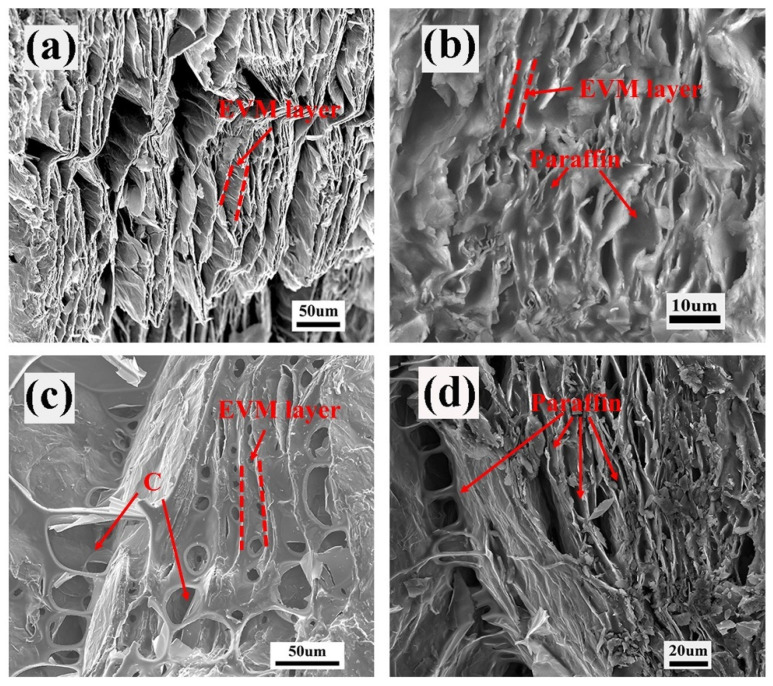
FESEM pictures of (**a**) EVM, (**b**) EVMP, (**c**) EVMC, and (**d**) EVMCP.

The morphological characteristics of EVM, EVMP, EVMC, and EVMCP are shown in Figure 7. It is apparent from Figure 7a that the expanded vermiculite had a rich layer/gap structure, which can provide abundant storage space for paraffin and ensure that paraffin had high morphological stability. Figure 7c shows that carbon layers were grown in situ on the surface of the expanded vermiculite upon carbonization and that the porous carbon structure was created in the EVM layers. Therefore, the porosity of EVMC increased after carbonization and it possessed a robust surface tension and capillary force, which had good adsorption performance for paraffin. Figure 7a,c respectively show that the pores of EVM and EVMC were almost completely filled with paraffin after vacuum impregnation. In addition, Figure 7c further illustrates that paraffin was first adsorbed by the pore walls of the porous carbon. It shows that the paraffin was not completely filled into the pores of the porous carbon but evenly covered the pore walls of the porous carbon, and thereby increased the thickness of the pore walls. From the perspective of the filling of paraffin, although there was still room to further increase the load, this incomplete filling state also prevented the leakage during the phase change process. It suggested that paraffin had been successfully adsorbed in the interlayer pore of EVM and EVMC.

### 3.6. Thermal Stability Analysis of ss-CPCMs Based on EVM

**Figure 8:**
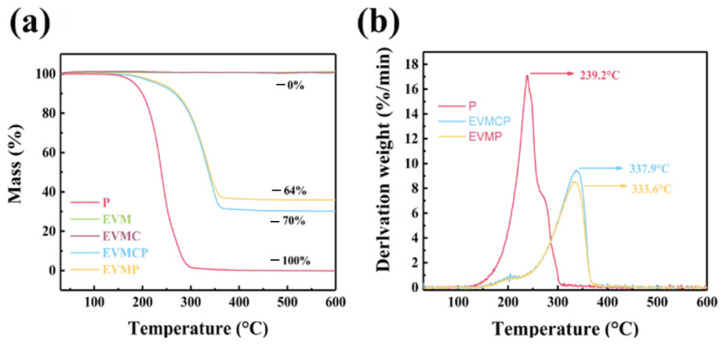
(**a**) TGA curves and (**b**) DTG curves of Paraffin, EVM, EVMC, and the ss-CPCMs.

The stability analysis of paraffin, EVM, EVMC, and the ss-CPCMs based on EVM were represented by TG and DTG are shown in Figure 8.

In the test temperature range below 600 °C, the paraffin completely decomposed, while the matrix had no mass loss at 600 °C, so the weight loss of EVMP and EVMCP was 64 wt% and 70 wt%, respectively, which could be attributed to the weight loss of the paraffin. In addition, this was consistent with the paraffin content obtained by DSC. The thermogravimetric results further indicated that paraffin was successfully adsorbed in the EVM and EVMC matrices, and the adsorption capacity of EVMC to paraffin was further enhanced compared with EVM. The maximum decomposition temperature delay of EVMP and EVMCP was 333.6 °C and 337.9 °C, respectively. The results showed that in EVM and EVMC carrier materials, the decomposition of paraffin was delayed, and the slowing effect of EVMC was better, indicating that EVMCP composite phase change energy storage materials had better thermal stability.

### 3.7. Thermal Conductivity of the ss-CPCMs

**Figure 9:**
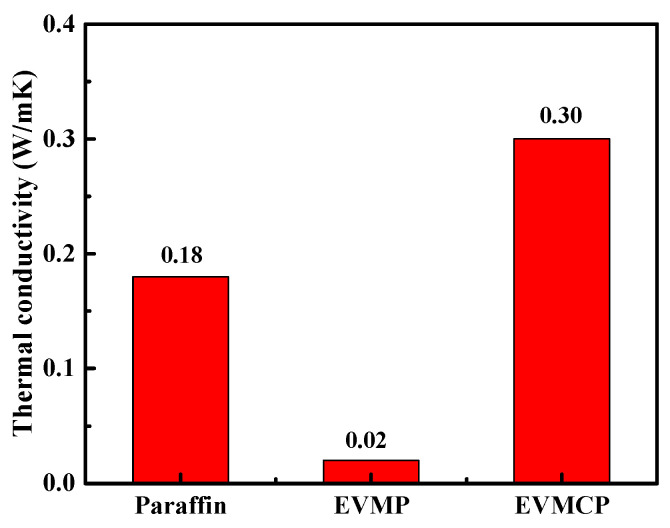
Thermal conductivities of Paraffin, EVMP, and EVMCP.

DSC was utilized to measure the specific heat (*C_P_*), whereas the LFA was employed to ascertain the thermal diffusivity (α) at a specific temperature. To calculate the thermal conductivity (*γ*) of paraffin, EVMP, and EVMCP, the Equations (1)–(3) were utilized. This is demonstrated in Figure 9, which can be found below:

(1) $$V=\pi r^{2}h$$

(2) $$\rho =\frac{M}{V}$$

(3) $$\gamma =\alpha \times \rho \times C_{P}$$

where *V* and ρ stood for the observed sample’s volume and density, respectively, and *γ* and h for its radius and thickness.

The findings demonstrated that paraffin and EVMP have thermal conductivities of 0.18 and 0.02 W/m K, respectively. It implied that because of EVM’s poor heat conductivity, and there are still unfilled parts inside the EVMP during the paraffin encapsulation process, resulting in poor heat transfer. Compared with Paraffin and EVMP, the thermal conductivity of EVMCP was 0.30 W/m K, and its thermal conductivity was 66.7% higher than that of P, showing that the heat transfer capability was greatly improved. In the enlarged vermiculite pore structure, porous carbon creates rich three-dimensional heat transfer networks and heat transfer channels. By increasing the heat transfer channel and efficiently lowering the thermal resistance, the pore structure of EVMC improved heat transfer. Compared with ordinary particle doping, this strategy had more advantages in thermal conductivity [44,45].

### 3.8. Non-Isothermal Crystallization Kinetics of the ss-CPCMs

**Figure 10:**
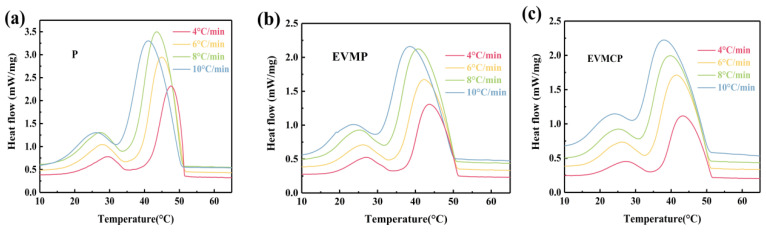
Non-isothermal crystallization DSC thermograms of (**a**) Paraffin (P), (**b**) EVMP, (**c**) EVMCP at different cooling rates.

Figure 10 illustrates the non-isothermal crystallization DSC curves of paraffin, EVMP, and EVMCP at different cooling speeds (φ). Table 3 enumerates the onset temperature (*T_O_*), peak temperature (*T_P_*), and end temperature (*T_E_*). The transition point of P shifted to lower temperatures as the cooling rate intensified. Moreover, the exothermic DSC curve exhibited an increased breadth, accompanied by more pronounced exothermic peaks. The paraffin molecular chain lacked sufficient time to finalize movement and rearrangement during the transition from amorphous to crystalline at an accelerated cooling rate. This behavior was observable in the prepared ss-CPCMs.

The relative crystallinity (*X_t_*) of EVMP and EVMCP were examined in order to look into their non-isothermal crystallization behavior. The following Formula (4) [46] can be used to determine the relative crystallinity (*X_t_*), which was related to the ratio of latent heat (*H*) from *T_O_* to *T* and *T_O_* to *T_E_*. Furthermore, Formula (5) can be used to define the crystallinity time (*t*), where cooling rate was denoted by φ.

(4) $$X_{t}=\frac{\int_{T_{O}}^{T}(\frac{dH}{dT})dT}{\int_{T_{O}}^{T_{E}}\left(\frac{dH}{dT}\right)dT}\times 100\%$$

(5) $$t=\frac{T_{0}-T}{\varphi }$$

**Figure 11:**
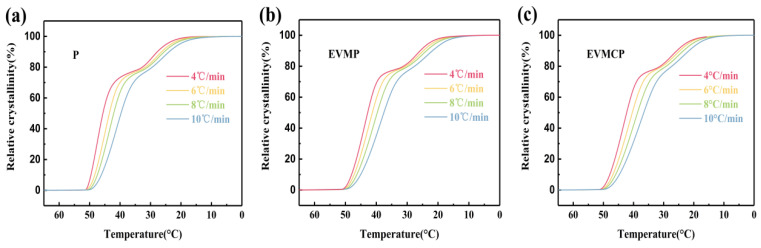
Relative crystallinity as a function of crystallization temperature of (**a**) Paraffin (P), (**b**) EVMP, (**c**) EVMCP at different cooling rates.

Figure 11 illustrates the correlation between temperature (T) and relative crystallinity (*X_t_*) for P, EVMP, and EVMCP. The relative crystallinity of P, EVMP, and EVMCP rapidly developed as the temperature dropped. At the same relative crystallinity, the crystallization temperatures dropped as the cooling speeds rose. Furthermore, the crystallization temperatures decreased with increasing cooling speeds. In this instance, nucleation often controlled the crystal’s phase transition.

**Figure 12:**
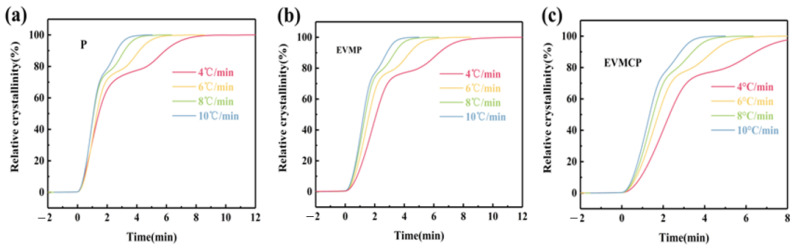
Relative crystallinity as a function of crystallization time of (**a**) Paraffin (P), (**b**) EVMP, (**c**) EVMCP at different cooling rates.

Figure 12 shows the correlation between time (*t*) and relative crystallinity (*X_t_*). When the crystallinity was 50%, the corresponding time was known as the half crystallization time (T_1/2_), and Table 3 listed the values of t_1/2_. As can be observed from Figure 11 and the half crystallization time (T_1/2_) data, the value of T_1/2_ of ss-PCM was higher than the P at the same rate. This showed that under identical conditions, the crystallization speed of ss-PCM was slower. This could be because the packing matrix’s micro-nanopores had an inhibitory influence on P’s crystallization behavior.

**Figure 13:**
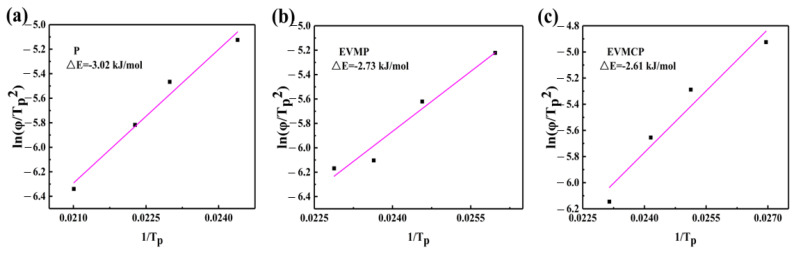
Plots of activation energy of (**a**) Paraffin (P), (**b**) EVMP, (**c**) EVMCP obtained from Kissinger’s equation.

The nucleation activation energy (ΔE*_a_*) could be used to better investigate how the matrix affected the crystallization behavior of paraffin (*P*) by reflecting the difficulty of crystal nucleation under non-isothermal crystallization circumstances. The ΔE*_a_* can be calculated using Kissinger’s equation (Equation (6)).

(6) $$ln\left(\frac{\varphi }{T_{P}^{2}}\right)=-\frac{{∆E}_{a}}{R}\times \frac{1}{T_{P}}+ln\left(\frac{A\times R}{∆E_{a}}\right)$$

where *R* was the gas constant and *A* was the frequency factor, and the value of ΔE*_a_* can be determined via the slope of the curve of ln(1/*T_P_*) relative to 1/*T_P_*, as shown in Figure 13.

The nucleation activation energy of P was −3.02 k J/mol. Compared with P, the nucleation activation energies of EVMP and EVMCP were significantly reduced (−2.73 k J/mol and −2.61 k J/mol, respectively). This shows that EVM and EVMC significantly accelerated the nucleation of P and reduced the energy barrier of heterogeneous nucleation, which was helpful for the nucleation process of P, demonstrating that it improved the crystallization behavior of ss-CPCMs to a certain extent.

**Figure 14:**
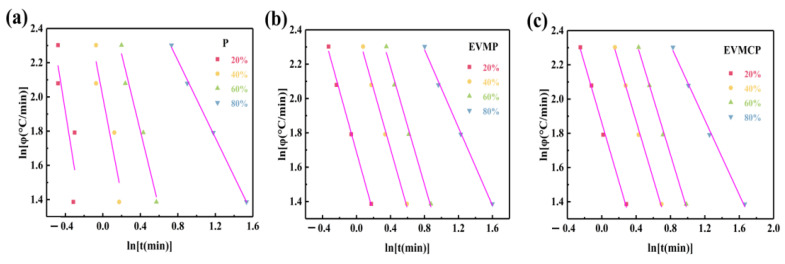
Plots of ln*φ* versus ln*t* of (**a**) Paraffin(P), (**b**) EVMP, (**c**) EVMCP (Xt = 20%, 40%, 60%, and 80%).

In order to better analyze the non-isothermal crystallization process, its crystal growth ability can be calculated using the joint equation of Avrami and Ozawa (defined by Equation (7)).

(7) $$lnZ_{t}+nlnt=lnK\left(T\right)-mln\varphi$$

where *n* and *m* represent the Avrami and Owaz indices, respectively. *K*(*T*) was the cooling crystallization function. Equation (2) can be further derived as Equation (8):

(8) $$ln\varphi =lnF\left(T\right)-alnt$$

where *a* = n/m and *F*(*T*) = [*K*(*T*)*Z_t_*]^(1/m), with the parameter *F*(*T*) = [*K*(*T*)/*Z_t_*]^(1/m) representing the cooling rate. *K*(*T*) denoted the cooling rate necessary to attain the specified relative crystallinity within a designated timeframe, indicating that *F*(*T*) possesses both physical and practical significance for the non-isothermal crystallization process. Figure 14 illustrates the correlation between *lnφ* and *lnt* at *X_t_* values of 20%, 40%, 60%, and 80%. The *F*(*T*) can be derived from the intercept of the linear fit, and the corresponding calculation data are organized in Table 4.

The results showed that the value of *F*(*T*) increases with the increase of relative crystallinity, indicating that the faster the cooling rate, the more difficult it was to crystallize. In addition, the value of *F*(*T*) of EVMP and EVMCP were higher than that of P under the same *X_t_*, revealing that the crystallization rate of EVMP and EVMCP were lower. It showed that the non-isothermal crystal growth of P was restricted by the interaction of the EVM and EVMC’s surface.

In conclusion, EVM and EVMC were useful in further lowering P’s nucleation activation energy and making it easier for P to nucleate. However, it continued to hinder the crystal formation process of P to varying degrees.

### 3.9. Cyclic Stability Analysis of the Composite PCM

**Figure 15:**
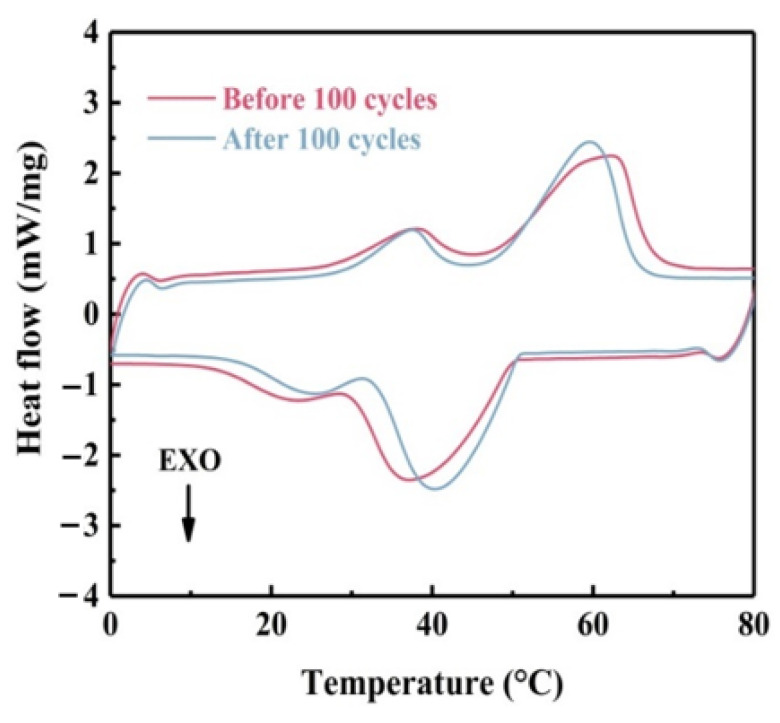
DSC curves of EVMCP ss-CPCMs before and after 100 melting and solidification cycles.

**Figure 16:**
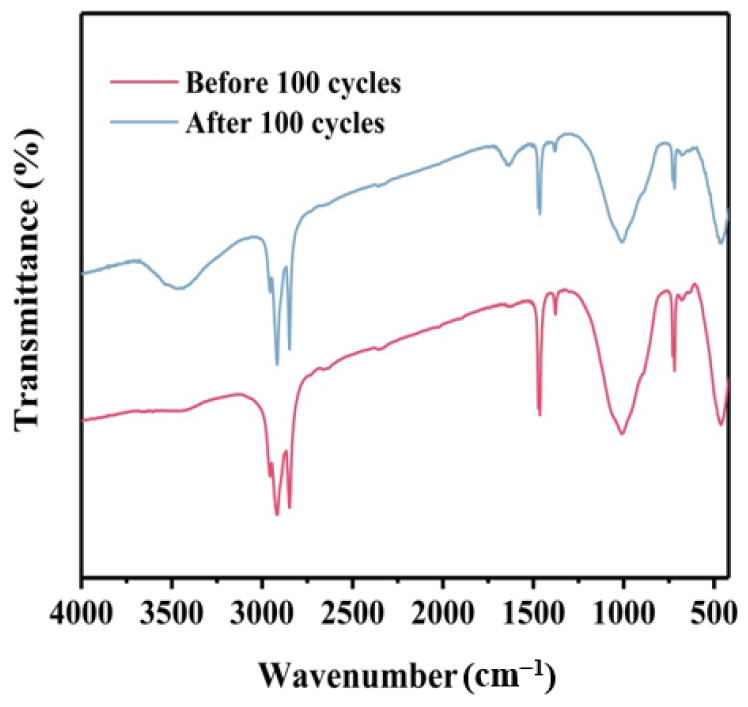
FT-IR spectra of EVMCP ss-CPCMs before and after 100 melting and solidification cycles.

Cycle stability was an important consideration for composite phase change materials. The DSC curves of the shape-stable composite PCM before and after 100 thermal cycles are shown in Figure 15 and the DSC data are listed in Table 5. The DSC curves before and after the cycle were practically identical. The thermal parameters did not change much, the values of T_m_ and T_f_ of the composite phase change material were 62.2 °C and 37.1 °C, respectively, before the cycle, and became 59.5 °C and 40.3 °C after the thermal cycle. The latent heat dropped slightly from 179 J/g to 170.3 J/g. The infrared spectrum after 100 cycles of EVMCP is shown in Figure 16. It was evident that EVMCP can continue to exhibit excellent chemical stability following the cycle because the location of the distinctive peaks of the prepared EVMCP was constant before and after the cycle. This was attributed to the closely interconnected multiphase interface among paraffin, EVM, and porous carbon, which endowed the composite phase change material with excellent cycling performance. As a result, the developed EVMCP composite phase change material exhibited excellent heat cycle stability.

## 4. Conclusions

In the study, the porous carbon was grown in situ in the vermiculite layers, and then the paraffin was encapsulated to prepare CPCM, and its thermophysical properties were analyzed. The following conclusions are presented below.

The MIP data showed that the porosity of EVMC was 89.92%, which was higher than that of EVM at 82.31%. As a result, the encapsulation rate of paraffin by EVMC was 28.8% higher than that by EVM. This reflected that starch-modified EVM enhanced the multistage pore structure of EVM, while the starch-based porous carbon framework acted as a “bridge” connecting adjacent layers of EVM, which stabilized the double-solid sealing phase variable material.

The thermal conductivity of EVMCP was 0.30 W/m K, which was 66.7% higher than paraffin. This indicated that the network structure of porous carbon-marked paraffin, EVM, and porous carbon forms a multi-phase interface, which increased the bonding tightness of EVMC’s pore structure skeleton and paraffin at the interface, thereby reducing the interface thermal resistance and improving the heat exchange rate.

Furthermore, The DSC data showed that EVMCP exhibited a higher latent heat of 179.0 J/g, which represented an increase of 23.6% compared to EVMP. Based on the activation energy and non-isothermal crystallization dynamics of EVMP and EVMCP, it is possible to conclude that EVM and EVMC increased paraffin nucleation while limiting crystal development. As a result, due to the construction of a carbon framework, the encapsulation efficiency of phase change materials was improved and the degree of undercooling was reduced. This provided a reference for matrix modification in the field of phase change.

## Figures and Tables

**Table 1:** Pore structure parameters of EVM and EVMC.

Samples	Average Pore Diameter (μm)	Specifiic Pore Volume (mL/g)	Porosity (%)
EVM	1.11	2.83	82.31
EVMC	1.21	3.92	89.82

**Table 2:** EVM-based phase change parameters for paraffin and ss-CPCMs.

Samples	Melting Process		Solidification Process		$\eta =\frac{H_{theo}}{H_{pcm}}$
	T_M_ (℃)	H_M_ (J/g)	T_s_ (°C)	H_s_ (J/g)	
Paraffin	36.7/58.2	247.2	28.0/43.8	259.1	——
EVMP	37.4/60.6	144.8	23.7/38.5	157.6	59%
EVMCP	38.1/62.2	179.0	23.3/37.1	179.2	72%

**Table 3:** Crystallization latent heat, temperature, and kinetic parameters of Paraffin, EVMP, and EVMCP under non-isothermal conditions.

Samples	Φ (℃/min)	*T_O_* (℃)	*T_p_* (℃)	*T_E_* (℃)	*T*_1/2_ (min)	ΔE*_a_* (kJ/mol)
P	4	51.4	47.6	41.4	1.43	−3.02
	6	51.4	44.9	38.7	1.32	
	8	50.8	43.5	36.7	1.09	
	10	50.4	41	34.2	1.07	
EVMP	4	51.2	43.7	37.9	2.11	−2.73
	6	51	42.3	35.4	1.62	
	8	50.6	40.7	33.2	1.37	
	10	50	38.5	30.3	1.26	
EVMCP	4	51.5	43.2	37	2.30	−2.61
	6	51	41.4	34	1.76	
	8	50.8	39.8	31.5	1.51	
	10	50	37.1	28.6	1.34	

**Table 4:** Comparison of the values of *F*(*T*) of Paraffin, EVMP, and EVMCP at different relative crystallinity.

Samples	*X_t_* (%)	*F*(*T*) (K/min)
P	20%	1.7
	40%	7.4
	60%	14.9
	80%	22.6
EVMP	20%	5.4
	40%	11.1
	60%	17.6
	80%	24.4
EVMCP	20%	6.4
	40%	12.8
	60%	20.1
	80%	24.2

**Table 5:** Thermal characteristics of the EVMCP before and after 100 thermal cycles.

Samples	Melting Process		Solidification Process	
	T_M_ (℃)	H_M_ (J/g)	T_s_ (℃)	H_s_ (J/g)
EVMCP (Before cycles)	38.1/62.2	179.0	23.3/37.1	179.2
EVMCP (After cycles)	37.6/59.5	170.3	22.7/40.3	178.3

## Data Availability

The original contributions presented in the study are included in the article, further inquiries can be directed to the corresponding author.
