# Correction: Chen et al. Effects of In Situ Porous Carbon Modification on Thermal Energy Storage of Paraffin/Expanded Vermiculite Form-Stable Composite Phase Change Materials. *Materials* 2025, *18*, 870

**DOI:** 10.3390/ma18163881

**Published:** 2025-08-19

**Authors:** Huijing Chen, Shaogang Zhang, Yixiu Xin, Jiaqing Zhao, Jinhong Li, Xin Min, Xiaoguang Zhang

**Affiliations:** 1 Beijing Key Laboratory of Materials Utilization of Nonmetallic Minerals and Solid Wastes, National Laboratory of Mineral Materials, School of Materials Science and Technology, China University of Geosciences, Beijing 100083, China; 2 College of Materials Science and Engineering, Beijing University of Technology, No. 100 Pingleyuan Street, Chaoyang District, Beijing 100124, China

In the original publication [1], there was an error regarding the order of authors. Huijing Chen is now listed as the first author, and Shaogang Zhang as the second author. The corrected Author Contributions statement appears here. The authors state that the scientific conclusions are unaffected. This correction was approved by the Academic Editor. The original publication has also been updated.
